# Cardiac inflammatory myofibroblastic tumor: does it recur after complete surgical resection in an adult?

**DOI:** 10.1186/1749-8090-7-44

**Published:** 2012-05-04

**Authors:** Xuedong Yang, Cangsong Xiao, Mei Liu, Yu Wang

**Affiliations:** 1 Institute of Geriatric Cardiology, Chinese PLA General Hospital, Beijing, People’s Republic of China; 2 Department of Cardiac Surgery, Chinese PLA General Hospital, Beijing, People’s Republic of China; 3 Department of Pathology, Chinese PLA General Hospital, Beijing, People’s Republic of China

**Keywords:** Inflammatory myofibroblastic tumor, Heart, Surgery, Prognosis

## Abstract

Inflammatory myofibroblastic tumor is currently considered to be a low-grade neoplasm, and it rarely involves the heart. We reported a rare case of a 59-year-old female who received cardiac surgery for complete resection of inflammatory myofibroblastic tumor in the left atrium. Five months after surgery, the patient presented with acute cardiogenic pulmonary edema and subsequent sudden death due to a left atrial tumor which protruded into the left ventricle through mitral annulus during diastole. The recurrence of inflammatory myofibroblastic tumor in the left atrium was strongly suggested clinically.

## 

Dear Editor,

Inflammatory myofibroblastic tumor (IMT) occurs mostly in children and young adults [1] and usually involves the lungs and gastrointestinal tract; it rarely involves the heart. Since the first report of the cardiac involvement of IMT in 1975 [2], there have been only a few additional reports, and sudden unexpected death due to cardiac IMT has rarely been reported in the medical literature [3,4]. When in the heart, IMT are polypoid masses with little myocardial infiltration, and there seems to be no particular site predilection in the heart other than endocardial location. Since cardiac IMT may be potentially fatal if a cardiac valve or the coronary arteries are involved, whenever feasible, complete surgical resection of the tumor remains the mainstay of treatment and seems to have a satisfactory outcome. However, the possibility of recurrence and the long-term prognosis after cardiac surgery have not been determined due to the rarity of these lesions and to the scant knowledge of their pathogenesis.

A 59-year-old Chinese female was hospitalized because of exertional dyspnea for 2 weeks. Transthoracic echocardiography showed a left atrial mass (Figure 1A). Coronary angiography revealed normal coronary arteries. She underwent cardiac surgery for resection of a 6.0 × 4.0 × 2.2 cm mass in the left atrium, which originated from the posterior wall of the left atrium between pulmonary vein ostia. Histological examination of the resected mass showed spindle cells and stroma infiltrated with abundant lymphocytes and plasma cells. Immunohistochemistry studies demonstrated positive for vimentin and smooth muscle actin, and negative for CD31, CD34 and desmin in spindle cell components (Figure 2), which was consistent with IMT. One week after cardiac surgery, echocardiography showed no mass in the left atrium. After discharge, she did not receive medical follow-up. Five months later, she presented to the emergency department with complaint of sudden onset of orthopnea and wheezing. She was rehospitalized for acute pulmonary edema. Bedside transthoracic echocardiography revealed a left atrial mass (3.2 × 3.1 cm) attached to the left atrial wall, which protruded into the left ventricle through mitral annulus during diastole and back to the left atrium during systole (Figure 1B). No pericardial effusion, left ventricular hypertrophy, or left ventricular segmental wall motion abnormalities were revealed. The ejection fraction was normal. Cardiac surgery for resection of the left atrial mass was scheduled. However, the patient was found unresponsive the day after rehospitalization. She was resuscitated but the attempt was unsuccessful. She died in the hospital 22 hours after rehospitalization. The relatives of the patient refused autopsy.

**Figure 1:**
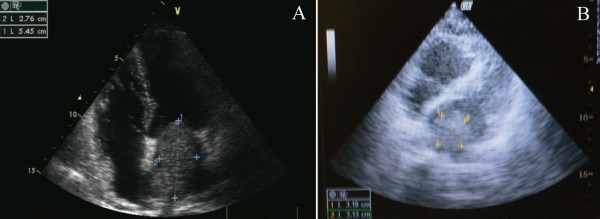
Transthoracic echocardiography revealed a left atrial mass (5.5 × 2.8 cm) before cardiac surgery (A), and a left atrial mass (3.2 × 3.1 cm) five months after surgical resection (B).

**Figure 2:**
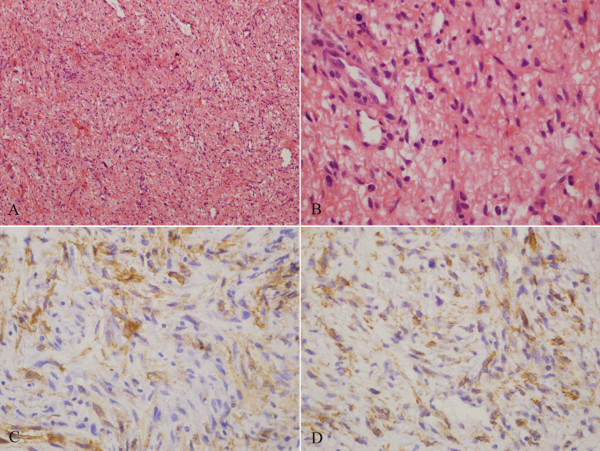
**Histological examination of the resected mass in the left atrium showed bland spindle cells and stroma infiltrated with abundant lymphocytes and plasma cells (A; H&E × 100); spindle cells have mild atypia with rare mitosis (B; H&E × 400).** The spindle cells are positive for smooth muscle actin **(C)** and vimentin **(D)** immunostain.

Up to now, the pathogenesis of IMT remains unclear, although it is thought to be an abnormal and exaggerated immunologic response by proliferated spindle cells and primary myofibroblasts to injury, inflammation, and infection. The diagnosis of IMT can be difficult due to the wide morphological spectrum. Coffin et al. have classified the histological findings of extrapulmonary IMT into three patterns: (a) spindle cells in a myxoid background with a vascular and inflammatory component resembling nodular fasciitis (myxoid/vascular pattern); (b) compact spindle cells with intermingled inflammatory cells resembling fibrous histiocytoma (compact spindle cells pattern); and (c) dense plate-like collagen resembling a desmoid or scar (hypocellular fibrosis pattern) [5]. In the present case, the histological findings of the resected cardiac mass presented as the myxoid/vascular pattern. Most of the spindle cells have the characteristics of myofibroblasts, and the immunohistochemical profile of reaction with vimentin in 99 % and smooth muscle actin in 92 % is typical of this phenotype [6]. The differential diagnosis of IMT comprises spindle cell sarcoma and inflammatory fibrosarcoma. Sarcoma is the most common primary malignant tumor of the heart. In IMT, spindle cells lack the cytologic atypia and nuclear hyperchromasia of sarcoma, and the immunohistochemical profile of myofibroblasts help to rule out spindle cell sarcoma. It can be difficult to distinguish IMT from inflammatory fibrosarcoma; both are included in the same section in World Health organization classification. Inflammatory fibrosarcoma is a locally aggressive, potentially metastasizing myofibroblastic tumor that occurs predominantly in the mesentery and retroperitoneum of children and young adults [7,8]. Inflammatory fibrosarcoma is now considered indistinguishable from, and within the morphological spectrum of, IMT [9]. Since cardiac involvement of inflammatory fibrosarcoma has not been reported, cardiac IMT was diagnosed in the present case.

IMT is considered a low-grade neoplasm. The recent classification of the World Health Organization recognized the uncertainty of its biological nature, that is, its clinical features might resemble malignant neoplasia. Whenever feasible, complete surgical resection remains the mainstay of treatment. Up to now, the recurrence rate of pulmonary IMT is low. However, local recurrence rate of extrapulmonary IMT is up to 25 % [6]. To the best of our knowledge, only one case of recurrent cardiac IMT has been reported [10]; IMT recurred at the right ventricular outflow tract in a 5-month-old infant. In the present case, the 59-year-old patient presented with acute pulmonary edema and subsequent sudden death due to a left atrial tumor five months after complete surgical resection of IMT in the left atrium. The left atrial tumor extended into the left ventricle through the mitral annulus, causing obstruction at the left ventricular inlet followed by acute pulmonary edema and decreased cardiac output. In spite of lack of autopsy evidence, the recurrence of left atrial IMT was strongly suggested clinically. The present case suggested that, although cardiac IMT was usually thought to be low-grade neoplasm and generally did not recur after complete surgical resection, given the potential catastrophic sequel of cardiac IMT, it was strongly recommended that the patients should be closely followed up including regular echocardiography, even if they were asymptomatic after surgical resection of cardiac IMT.

## Abbreviations

IMT = inflammatory myofibroblastic tumor.

## Authors’ contribution

YX collected data and drafted the manuscript. XC carried out the literature research. LM was helpful in literature review. WY conceived the study and critically revised the manuscript.

## Competing interests

The authors declare that they have no competing interests.
